# Early Quick Acuity Score Provides More Complete Data on Emergency Department Walkouts

**DOI:** 10.1371/journal.pone.0085776

**Published:** 2014-01-17

**Authors:** Paris B. Lovett, J. Akiva Kahn, Stuart E. Greene, Matthew A. Bloch, Daniel R. Brandt, Michael R. Minckler

**Affiliations:** 1 Department of Emergency Medicine, Thomas Jefferson University, Philadelphia, Pennsylvania, United States of America; 2 Department of Emergency Medicine, Providence Little Company of Mary Hospital, Torrance, California, United States of America; 3 Emergency Medicine Residency, Thomas Jefferson University Hospital, Philadelphia, Pennsylvania, United States of America; 4 Coordinating Center for Clinical Research, Thomas Jefferson University, Philadelphia, Pennsylvania, United States of America; 5 Thomas Jefferson University, Philadelphia, Pennsylvania, United States of America; University Medical Center Utrecht, Netherlands

## Abstract

**Introduction:**

Many prior studies have compared the acuity of Emergency Department (ED) patients who have Left Without Being Seen (LWBS) against non-LWBS patients. A weakness in these studies is that patients may walk out prior to the assignment of a triage score, biasing comparisons. We report an operational change whereby acuity was assessed immediately upon patient arrival. We hypothesized more patients would receive acuity scores with EQAS. We also sought to compare LWBS and non-LWBS patient characteristics with reduced bias.

**Methods:**

Setting: urban, academic medical center. Retrospective cohort study, electronic chart review, collecting data on all ED patients presenting between 4/1/2010 and 10/31/2011 (“Traditional Acuity Score” period, TAS) and from 11/1/2011 to 3/31/2012 (“Early Quick Acuity Score” period, EQAS). We recorded disposition (LWBS versus non-LWBS), acuity and demographics. For each subject during the EQAS period, we calculated how many prior ED visits and how many prior walkouts the subject had had during the TAS period.

**Results:**

Acuity was recorded in 92,275 of 94,526 patients (97.6%) for TAS period, and 25,577 of 25,760 patients (99.3%) for EQAS period, a difference of 1.7% (1.5%, 1.8%). LWBS patients had acuity scores recorded in 5,180 of 7,040 cases (73.6%) during TAS period, compared with 897 of 1,010 cases (88.8%) during the EQAS period, a difference of 15.2% (14.8%, 15.7%). LWBS were more likely than non-LWBS to be male, were younger and had lower acuity scores. LWBS averaged 5.3 prior ED visits compared with 2.8 by non-LWBS, a difference of 2.5 (1.5, 3.5). LWBS averaged 1.3 prior ED walkouts compared with 0.2 among non-LWBS, a difference of 1.1 (0.8, 1.3).

**Conclusions:**

EQAS resulted in a higher proportion of patients receiving acuity scores, particularly among LWBS. This offers more complete data when comparing LWBS and non-LWBS patient characteristics. The comparison reinforced findings from prior studies.

## Introduction

### Background

Patients who leave EDs before being assessed by a healthcare provider have been a major area of research and discussion over the past three decades[1].

A key concern is that high acuity patients may be walking out, at great risk to their health. If patients walk out before an acuity score is assigned, data on this issue will be incomplete. Prior reports comparing acuity between LWBS and non-LWBS patients have suffered a common structural flaw: if failure to record an acuity score does not occur at similar rates between groups, and in a random fashion, such comparisons will potentially be biased.

In a traditional ED workflow, ambulatory patients are greeted first by non-clinical staff, who collect identification and basic demographic information, and then subsequently are seen by nurses who perform a triage assessment, collect detailed information on the chief complaint, and assign an acuity score such as the Emergency Severity Index (ESI). Ideally, triage and assignment of acuity score will occur within a few minutes of presentation, but it is not uncommon for longer waits to occur. During this interval, patients may walk out before acuity scores are assigned.

We report on a new workflow approach in which the first person encountered by ambulatory patients is an emergency nurse (EN) who collects the initial demographics, and simultaneously assigns a quick ESI score (Early Quick Acuity Score, EQAS), using an abbreviated process.

We hypothesized that recording an acuity score at an earlier stage in the patient encounter would lead to an increase in the percentage of patients with ESI acuity scores assigned, and that the increase would be particularly marked among LWBS patients.

Further, if we could demonstrate an increase in the percentage of walkout patients assigned ESI scores, then we could also provide an improved set of comparisons between LWBS and non-LWBS patients, looking at acuity and demographics.

Finally, we sought to examine the association between LWBS and frequency of past ED visits, and past ED walkouts. We hypothesized that among LWBS patients the average number of prior visits, and the average number of prior walkouts, would be higher than among non-LWBS patients.

## Materials and Methods

### Study Design and Setting

Large, urban academic medical center with an EM residency program, and approximately 63,000 annual visits. We conducted a retrospective cohort study based on review of emergency department electronic medical records. An Electronic Medical Record (EMR) was introduced in March 2010. An Early Quick Acuity Score (EQAS) was introduced November 1, 2011.

### Selection of Participants

We abstracted data for all patient encounters between 4/1/2010 and 10/31/2011 (“Traditional Acuity Score”, TAS period) and from 11/1/2011 to 3/31/2012 (EQAS period).

### Interventions

Prior to the introduction of the EQAS protocol, this ED had a traditional triage process consisting of the immediate collection of demographic data, followed by triage and the assignment of the Emergency Severity Index (ESI) score in a traditional manner, occurring after a brief wait.

In July 2011 we introduced a new position known as “First Nurse.” The First Nurse replaced the non-clinical registrar as the person first making contact with new ambulatory ED patients. Acuity scores continued to be assigned in the traditional manner, during formal triage.

On November 1, 2011 the First Nurse began assigning acuity scores at the same time as collecting initial demographics, thereby combining the initial registration function with acuity assessment. The First Nurse performed an ESI assessment in more rapid fashion based upon five data points: (1) mode of arrival (2) age (3) sex (4) chief complaint (5) visual appearance. This EQAS process employed the same conceptual algorithm[2] for assigning ESI as is used for traditional ESI. This algorithm involves considerations of whether the patient may be dying, should not wait to be seen, and how many resources the patient will require. The difference between TAS and EQAS is that the quicker EQAS approach relies upon fewer data points, is collected in a briefer period of time, and does not utilize vital signs. The aim in implementing this process was to perform an acuity assessment at an earlier stage in the patient visit and upon a higher proportion of patients, thus maximizing the opportunities to recognize higher acuity patients early in their ED visit. This was judged to be worth the tradeoff of having fewer data points on which to base the score. Flexibility in implementation structure is part of the ESI model.

Other operational changes were introduced during the same period, all aimed at improving patient throughput in the ED. These included placing an attending physician in the waiting room at times of peak demand, immediate placing of patients in beds when beds were available, use of vertical space (chairs) to increase capacity in the ED, and expansion of fast track to include higher acuity (ambulatory ESI 3) patients.

This study was determined to be exempt by the Office of Human Research, Division of Human Subjects Projection Institutional Review Board of Thomas Jefferson University (“IRB”). The IRB approved a waiver of written consent by the patients, and/or the next of kin, caretakers, or guardians on the behalf of the minors/children participants, for their information to be stored in the hospital database and used for research.

### Methods and Measurements

We classified patient visits into two time periods. The TAS period was from April 1, 2010 until October 31, 2011. The EQAS period was from Nov 1, 2011 until March 31, 2012.

Data was extracted directly from the EMR by the primary investigator using an automated pre-existing standardized report. Data was exported as a spreadsheet file (Microsoft Excel 2010, Microsoft Corp., Redmond, Washington) from the EMR software, then imported into a statistical analysis program (StataCorp. 2009. Stata Statistical Software: Release 11. College Station, TX: StataCorp LP).

The STROBE checklist for observational studies[3] guided presentation of the study methodology and findings.

### Outcomes

For each patient we abstracted age, sex, race/ethnicity, disposition (walkout, elopement, Against Medical Advice (AMA), admitted, discharged, expired, transferred) and ESI. Elopement refers to patients who leave after being seen by a provider, without signing an “Against Medical Advice” form. AMA refers to patients who leave after being seen by a provider, and who sign an “Against Medical Advice” form.

Insurance status was not abstracted, as it is not collected during the First Nurse process. For patients during the EQAS period, we also performed a lookup function for prior ED visits by the same individual during the TAS period, and counted these prior visits, and specifically counted how many of these visits had a disposition of LWBS.

### Analysis

We compared data from the TAS period to the EQAS period, examining the following: percentage of LWBS patients who had an ESI assigned before walking out; percentage of all patients who had an ESI assigned before disposition.

We examined data from the EQAS period, comparing LWBS patients to non-LWBS patients, for the following characteristics: age, sex, race/ethnicity, number of prior visits during the TAS period, number of walkouts during the TAS period, and ESI scores.

One analysis involved comparing LWBS and non-LWBS patients during the EQAS period, to see whether they had different prior histories in terms of frequency of ED visits and frequency of walkouts. Thus, for each individual patient during the EQAS period, we calculated prior visits, and prior walkouts, by the same individual patient, during the (preceding) TAS period. We then compared EQAS period LWBS and non-LWBS patients, to see if the same individual patients had different rates of ED visits and walkouts during the TAS period.

Data analysis was performed in STATA 11 using the t-test for differences between means and the two-sample z-test for the differences between proportions.

Sample size calculations showed that an EQAS period with at least 8,000 patients and 700 walkouts would exceed a power of 0.8 to detect, respectively, a change of 0.5% in the proportion of all patients with ESI scores obtained, and to detect a change of 5% in the proportion of walkout patients with ESI scores obtained.

## Results

During the TAS period there were 94,526 ED patients, of whom 7,040 were LWBS. During the EQAS period there were 25,760 ED patients, of whom 1,010 were LWBS. Thus, the rate of walkouts dropped from 7.4% during the TAS period to 3.9% during the EQAS period, a difference of 3.5% (3.2%, 3.8%).

Table 1 shows patient characteristics, and a comparison of proportions of patients with acuity scores, between the TAS and EQAS periods.

**Table 1:** Comparison of TAS and EQAS periods: LWBS rates, acuity (ESI) assignment rates, demographics, acuity (ESI) scores.

	TAS		EQAS		Difference (95% CI)
**Characteristics**	N	(%)	N	(%)	
All	94,526		25,760		
LWBS	7,040	7.4%	1,010	3.9%	−3.5% (−3.8%, −3.2%)
**ESI Assigned (all)**	92,275	97.6%	25,577	99.3%	1.7% (1.5%, 1.8%)
**ESI Assigned (Non-LWBS)**	87,095	99.6%	24,680	99.7%	0.2% (0.1%, 0.2%)
**ESI Assigned (LWBS)**	5,180	73.6%	897	88.8%	15.2% (14.8%, 15.7%)
**Age**					
<18	4,439	4.7%	1,250	4.9%	
18-34	30,175	31.9%	8,341	32.4%	
35–49	24,057	25.5%	6,265	24.3%	
50–64	21,401	22.6%	5,752	22.3%	
65–79	9,373	9.9%	2,752	10.7%	
>79	4,703	5.0%	1,308	5.1%	
**Sex**					
Female	51,153	54.1%	13,929	54.1%	
Not Recorded	2	0.0%	3	0.0%	
**Race**					
Asian	3,006	3.2%	908	3.5%	
African-American	45,869	48.5%	12,437	48.3%	
Native American	240	0.3%	69	0.3%	
Hispanic	4,730	5.0%	1,434	5.6%	
White	37,183	39.3%	10,187	39.5%	
Other	238	0.3%	52	0.2%	
**ESI**					
1	508	0.5%	198	0.8%	
2	14,649	15.5%	3,880	15.1%	
3	50,980	53.9%	14,543	56.5%	
4	24,224	25.6%	6,426	24.9%	
5	1,914	2.0%	530	2.1%	
Not Recorded	2,251	2.4%	183	0.7%	

The percentage of LWBS patients who were assigned an ESI score before walking out increased from 5,180 out of 7,040 cases (73.6%) during the TAS period to 897 out of 1,010 cases (88.8%) during the EQAS period, a difference of 15.2% (14.8%, 15.7%). For all patients (LWBS and non-LWBS), acuity scores were recorded in 92,275 out of 94,526 patients (97.6%) for TAS period, and 25,577 of 25,760 patients (99.3%) for EQAS period, a difference of 1.7% (1.5%, 1.8%).

Table 2 shows data from the EQAS period only, with a comparison of non-LWBS and LWBS patients during that period. The table shows differences in the rates of ESI assignment between non-LWBS and LWBS. Additionally, it compares the prior visit history of individual patients from the EQAS period. Specifically, for each individual who visited during the EQAS period, we examined how many prior visits and prior walkouts the same individual had had during the TAS period.

**Table 2:** Data from the EQAS period only, with a comparison of non-LWBS and LWBS patients during that period.

	Non-LWBS		LWBS		Difference (95% CI)
**Characteristics**	N		N		
	24,750		1,010		
**ESI Assigned**	24,680	99.7%	897	88.8%	−10.9% (−12.9%, −9.0%)
**Avg ED Visits During TAS**		2.8		5.3	2.5 (1.2, 3.5)
**Avg ED LWBS During TAS**		0.2		1.3	1.1 (0.8, 1.3)
**Age**					
<18	1,194	4.8%	56	5.5%	0.7% (−0.7%, 2.2%)
18–34	7,983	32.3%	358	35.4%	3.2% (0.2%, 6.2%)
35–49	5,966	24.1%	299	29.6%	5.5% (2.6%, 8.4%)
50–64	5,517	22.3%	235	23.3%	1.0% (−1.7%, 3.6%)
65–79	2,699	10.9%	53	5.2%	−5.7% (−7.1%, −4.2%)
>79	1,301	5.3%	7	0.7%	−4.6% (−5.2%, −4.0%)
**Mean Age**		44.7		40.1	−4.6 (−5.6, −3.5)
**Sex**					
Female	13,488	54.5%	441	43.7%	−10.8% (−14.0%, −7.7%)
Not Recorded	3	0.0%	-	-	
**Race**					
Asian	889	3.6%	19	1.9%	−1.7% (−2.6%, −0.8%)
African-American	11,926	48.2%	511	50.6%	2.4% (−0.7%, 5.5%)
Native American	68	0.3%	1	0.1%	−0.2% (−0.4%, 0.0%)
Hispanic	1,391	5.6%	43	4.3%	−1.4% (−2.6%, −0.1%)
White	9,921	40.1%	266	26.3%	−13.7% (−16.5%, −11.0%)
Other	49	0.2%	2	0.2%	0.0% (−0.3%, 0.3%)
**ESI**					
1	198	0.8%	-	0.0%	−0.8% (−0.9%, −0.7%)
2	3,859	15.6%	21	2.3%	−13.3% (−14.3%, −12.3%)
3	14,033	56.9%	510	56.9%	0.0% (−3.1%, 3.1%)
4	6,134	24.9%	292	32.6%	7.7% (4.8%, 10.6%)
5	456	1.8%	74	8.2%	6.4% (4.7%, 8.1%)
Not Recorded	70	0.3%	113	12.6%	
**Mean ESI**		3.11		3.47	0.36 (0.31, 0.40)

The table shows differences in the rates of ESI assignment between non-LWBS and LWBS patients. Additionally, it compares the prior visit history of individual patients from the EQAS period. Specifically, for each individual who visited during the EQAS period, we examined how many prior visits and prior walkouts the same individual had had during the TAS period.

LWBS patients during the EQAS period showed a prior (TAS period) history of more frequent ED visits and more frequent walkouts than non-LWBS EQAS period patients. EQAS period LWBS patients averaged 5.3 prior (TAS period) ED visits compared with 2.8 prior ED visits by EQAS period non-LWBS patients, a difference of 2.5 (1.5, 3.5). EQAS period LWBS patients averaged 1.3 prior ED walkouts compared with 0.2 among EQAS period non-LWBS, a difference of 1.1 (0.8, 1.3). Walkouts constituted 32.3% of TAS period visits for EQAS period LWBS patients, versus 4.1% for non-LWBS EQAS period patients, a difference of 28.2% (25.3%, 31.1%). Thus there was an association among individual patients between higher ED utilization and walkouts, and an association between prior walkout activity and future walkouts.

Among patients in the EQAS period, the LWBS patients were more frequently male, and younger in age, when compared to non-LWBS patients. Acuity by all measures was lower among LWBS patients. Mean ESI was 3.47 compared to 3.11 among non-LWBS, a difference of 0.36 (0.31, 0.40). There were no ESI 1 (highest acuity) cases among LWBS patients. ESI 2 cases were dramatically lower at 2.3% among LWBS as compared with 15.6% among non-LWBS patients, a difference of 13.3% (12.3%, 14.3%).

## Discussion

LWBS is a focus of performance improvement in EM. There is important debate and research regarding just how much risk is involved in walking out, and who is most affected, and some of that is cited here. However, the view is commonly held that walkouts are a safety issue, that they represent an operational failure to match resources to demand, that they are a failure in service to patients, as well as a threat to the finances and growth prospects of the healthcare institution. Not only do EDs across the United States measure walkouts for these reasons, the Centers for Medicare & Medicaid Services (CMS) now publishes walkout data for the public as an important safety measure[4].

Thus it is important to understand LWBS patient characteristics such as acuity, demographics, and prior-visit patterns in order to understand the impact of LWBS upon patient safety and to potentially reduce LWBS percentages in the future. This study describes a novel approach to triage, with immediate acuity assessment, and its impact upon measurement of acuity among LWBS patients.

We have reported on a modification of the standard approach to assignment of acuity scores. Assigning an acuity score immediately upon arrival resulted in a higher percentage of patients overall receiving an ESI score, and that impact was particularly marked among patients who ultimately walked out.

There is inherent value in early assignment of acuity scores, through earlier recognition of, and prioritization of, higher acuity patients. Further, by measuring acuity at an early point in the patient encounter it is possible that we may have altered the likelihood that patients would walk out before being seen by a provider, thus contributing to the reduction in walkout rates between the TAS and EQAS period. However, as noted in the methods section, other operational changes were made at the same time as introduction of EQAS, with the goal of improving patient flow, so it is not possible to assess for a causal relationship between EQAS and the reduction in walkout rate between TAS and EQAS periods.

Even with the EQAS approach, a considerable number of walkout patients (183) did not receive ESI scores. This could happen in two ways – either the patient left in the middle of the very brief First Nurse process (after giving their name, but before completion of the EQAS process), or the First Nurse failed to record an acuity score. Such failures to record a score may reflect the fact that the First Nurse role and EQAS process were new to our institution, but also reflect the general fact that staff do not always complete all tasks assigned to them.

The EQAS approach has given us new information about the characteristics of LWBS patients. Prior studies comparing the acuity of LWBS with non-LWBS have been reported from systems with traditional triage structures, where the delay between initial contact and assignment of ESI scores allowed for some patients to walk out prior to a score being assigned.

It is worth considering here what we already know about LWBS patients. Prior research has documented characteristics of LWBS with remarkable consistency of findings, both in the United States and internationally[5]–[16]. These studies have reported that, compared with non-LWBS patients, LWBS patients are more likely to be young[5], [6], [9]–[11], [14], [16]–[18], male[14], [16], poor[5], [19], of minority race[5], [14], [20], non-English-speaking[5], [14], and either uninsured or on Medicaid[5], [12], [14], [17]–[20]. Acuity scores for LWBS are widely reported as lower than for non-LWBS[6], [9]–[12], [14]–[16], [18], [20], with rare exceptions[21]. Finally, one prior study reports that patients who have walked out previously were more likely to walk out again[17].

System factors reported as associated with LWBS include ED crowding and long wait times[5], [6], [11], [15], [18], [22]–[25], visits occurring during busier shifts[9], [11], [16], and EDs that are in county hospitals or trauma centers[19], teaching hospitals[19]–[21], urban locations[20], or high-volume EDs[14].

Walkouts present safety concerns that cannot be dismissed, despite the lower acuity scores reported among LWBS patients. Mortality after walkout is rare but does occur[8], with one large study showing mortality of 0.17% of LWBS patients within 48 hours, a rate almost the same as the mortality rate among non-LWBS patients in the same study[14]. Repeat visits to the ED after walking out are common[6], [11], [13], [14], [24], and admission from these repeat visits is not uncommon[6]–[8], [14], [24], [25]. Several reports have described a majority of walkouts seeking alternative care within one week[8], [13], [25].

Our study adds to this literature by increasing the response rate for acuity scores and thereby reducing potential bias in comparisons of acuity scores between LWBS and non-LWBS patients. Our results thus reinforce the prior studies with more complete data, showing lower acuity among LWBS patients. Our results are also in keeping with the prior studies in showing that LWBS patients are disproportionately young, male and have lower acuity scores.

A novel finding in this study is that patients who walked out during the EQAS period had a higher average number of prior visits during the TAS period than did non-walkouts. This suggests that patients who visit EDs more frequently are also more likely to walk out without being seen. We also saw that LWBS patients during the EQAS period had both higher absolute average numbers of prior walkouts and higher walkouts as percentage of prior visits, when compared with non-LWBS patients, consistent with the previously cited prior research[17]. This supports the intuitive hypothesis that people who have walked out in the past are more likely to walk out in the future.

## Limitations

This study was performed at a single institution, and results may not be fully applicable in other contexts.

As mentioned in the methods and discussion sections, introduction of EQAS was not the only operational change made between study periods. Other changes included placing an attending physician in the waiting room at times of peak demand, immediate placing of patients in beds when beds were available, use of vertical space (chairs) to increase capacity in the ED, and expansion of fast track to include higher acuity (ambulatory ESI 3) patients. These multiple operational changes were likely reflected in the reduced walkout rate seen in the EQAS period. While it is possible that these changes created potential biases and confounding factors, it does not seem that they would invalidate the key findings of this study. The difference in overall walkout rates, likely related to the operational changes between the TAS and EQAS periods, must be considered as a potential source of bias in this study.

The ESI is a widely used, validated approach to assigning acuity scores in EDs. It is designed to be implemented in a flexible manner, with different amounts of assessment time and data inputs being employed in different contexts[2]. The EQAS process we report here is consistent with that philosophy, but the assignment of acuity scores with more limited clinical input and particularly without vital signs has not been validated. There is, of course, a potential tradeoff between the quality of the acuity assessment and efficiency of attaining these assessments.

This study did not involve any form of clinical follow-up of patients beyond their ED visits, and so cannot contribute any data on mortality, morbidity, repeat ED visits, admissions or other important clinical outcomes.

When analyses were re-run by reviewers of this article using different statistical approaches (Chi-squared, Fischer's exact) some comparisons showed only borderline significance.

## Conclusions

Using an EQAS process resulted in a higher percentage of patients being assigned acuity scores. This was particularly true of patients who walked out prior to medical evaluation. The data coming from this approach offers a more complete comparison of acuity between LWBS and non-LWBS patients. Consistent with prior studies, LWBS patients had lower acuity scores than non-LWBS patients; specifically there were no ESI 1s and dramatically fewer ESI 2s. Also consistent with prior studies, LWBS patients were disproportionately younger and male. LWBS patients had prior histories of higher average numbers of ED visits, higher average numbers of walkouts, and a higher rate of walkouts among those prior visits than did non-LWBS patients. Future studies could examine whether there is a tradeoff with EQAS between quicker assessments and accuracy.
